# Mineral Materials as a Neutralizing Agent Used on Soil Contaminated with Copper

**DOI:** 10.3390/ma14226830

**Published:** 2021-11-12

**Authors:** Andrzej Cezary Żołnowski, Mirosław Wyszkowski, Elżbieta Rolka, Marta Sawicka

**Affiliations:** 1 Department of Agricultural and Environmental Chemistry, University of Warmia and Mazury in Olsztyn, Łódzki 4 Sq., 10-721 Olsztyn, Poland; andrzej.zolnowski@uwm.edu.pl (A.C.Ż.); elzbieta.rolka@uwm.edu.pl (E.R.); 2 Regional Inspectorate for Environmental Protection in Bialystok, Branch Office in Suwałki, ul. Piaskowa 5, 16-400 Suwałki, Poland; marsa_90@o2.pl

**Keywords:** copper, lime, clay, zeolite, phytotoxicity

## Abstract

The aim of the investigation was to evaluate the response of plants, using black mustard (*Brassica nigra* L. Koch) as a model plant, to soil contamination with copper (0, 200, 400, 600 mg Cu kg^−1^ of soil), and to determine the effectiveness of the Cu immobilization with mineral neutralizing materials, such as lime, clay and zeolite. The plant yield depended on soil contamination and mineral amendments. In the series without neutralizing materials, the level of 600 mg Cu kg^−1^ reduced the yield and increased leaf greenness. Lime alleviated the toxicity of Cu in objects with 200 mg Cu kg^−1^. Zeolite slightly mitigated the harmful effects of Cu at the level of 400 and 600 mg kg^−1^. Zeolite lowered the SPAD index. In the chemical composition of plants, the content of Cu, K, Mg, Na and Ca in plants increased to 400 mg Cu kg^−1^, while the content of P decreased to 600 mg Cu kg^−1^. Among the materials, lime reduced the Cu accumulation in plants the most, followed by clay. Cu narrowed the majority of ratios and widened the Ca:P and K:Ca ratios in plants. The applied mineral materials, except lime, did not significantly affect the formation of these indicators.

## 1. Introduction

The development of civilization is inextricably linked with environmental pollution. Dangerous factors of anthropogenic origin are involved in processes that intensify the migration of metals to the edaphotope, which contributes to the reduction of vegetation (devegetation) and water depletion (dehumidification) and is destructive to the agrophysical, physicochemical and biological properties of soils, providing them with toxic properties [1,2,3]. These transformations arise mainly from the deposition of heavy metals caused by the emissions of metal-bearing dust, liquid and solid industrial and municipal waste, agricultural fertilizers and plant protection products, and motor traffic flow through roads and streets [4,5,6,7,8,9,10,11,12]. Any metal (metalloid) can be considered an impurity if it is present where it is not desired or in a form or concentration that causes harmful effects to humans or the environment [13]. Metals are accumulated by soils to levels that become dangerous for higher plants and for soil microflora and microfauna [14].

Soil contamination with heavy metals occurs locally and mainly concerns industrialized areas. However, in some regions, in the vicinity of emission sources, soils show a significant degree of pollution [15,16]. The average Cu content in soils in the world is 38.9 mg Cu kg^−1^ DM [17]. In the vicinity of metallurgical plants, e.g., the Głogów Copper Smelter (Poland), the average concentration of Cu, depending on the depth of sampling, ranges from 2160 to 3230 mg Cu kg^−1^ [18]. Globally, soil contamination in the vicinity of smelters has reached 510–9700 mg Cu kg^−1^ (Sudbury, ON, Canada), 1400–3700 mg Cu kg^−1^ DM. (Coniston, ON, Canada) and 11600–14200 mg Cu kg^−1^ (Lubumbashi, DR-Congo) [19]. Moreover, soil contamination with copper may result from the unskillful and unbalanced application of pesticides [20,21,22]. An example may be the Bordeaux mixture, a fungicide based on copper compounds used until the end of the 19th century (CuSO_4_ + Ca(OH)_2_), and such forms of copper as Cu_2_O, Cu(OH)_2_, Cu(OH)_2_·CuCl_2_, CuSO_4_·3Cu(OH)_2_, used in viticulture, which led to the severe accumulation of Cu in soils of vineyards in France, Brazil, Croatia, and Spain. Cu concentrations in the 0–20 cm layer currently range from 332 to 560 mg Cu kg^−1^ soil [21,22]. Copper fungicides are also used in the protection of other agricultural plants [23,24]. The copper content in a 16-year-old Brazilian cocoa field was 993 mg Cu kg^−1^, which is 50 times greater than in a non-cocoa field. Copper contents in soils of British apple orchard soils have even reached 1500 mg Cu kg^−1^ [25].

Copper is not biodegradable [26], and in the soil can be dissolved, chelated, adsorbed by humus, bound by soil minerals, taken up and bound by soil microorganisms or higher organisms, and precipitated in the form of insoluble mineral compounds. The durability of these bonds depends on the soil pH, amount and type of minerals, redox potential, sorption capacity and organic matter content [7,27]. A method that allows reduction of the harmfulness of heavy metals is the addition of neutralizing materials to the soil [3,28,29,30] so as to bind metals in insoluble metal-mineral or organo-metallic forms, which—under favorable conditions—may remain in the soil in harmless forms for a long period [9,31]. The most common neutralizing materials are zeolites, bentonite, clay, lime, and also organic materials, like compost, tree bark, farmyard manure and peat [3,23,30]. Until recently, the methods of heavy metal immobilization have been the only available and feasible form of reclamation of contaminated soils, allowing the restoration of biological life and plant cover in degraded areas [29,32]. High concentrations of heavy metals in soil result in higher than natural concentrations in plants, which may result in an increased flow of metals to further links in the trophic chain [12,33], where they may accumulate and their harmful effects may be long-term. The excess of copper in the soil changes its properties [3] and thus modifies the chemical composition of plants, which results in a change in the ionic ratios between the elements. Too wide or too narrow ratios are very unfavorable in the case of forage plants [34,35,36]. Copper is an element whose deficiency is unfavorable for plants, but its excess may have a morphological, physiological, biochemical and molecular phytotoxic effect [20]. The excess of Cu interferes with various metabolic processes, which results in the disturbance of plant growth and development [20,33]. Losses caused by Cu in the growth and yield of food crops are much higher than the sum of other causes threatening food safety. Since soil contamination with Cu can also affect agricultural land, it is important to conduct research aimed at this complex problem, which has not been fully clarified [20].

Therefore, a research hypothesis was formulated that mineral materials used on Cu-contaminated sites can reduce the toxicity of copper to plants. This action would improve the yield and change the chemical composition of the mass obtained with the simultaneous diversification of the ionic ratios vs. the null hypothesis that there was no such interaction.

The aim of the investigation was to evaluate the response of plants, using black mustard (*Brassica nigra* L. Koch) as a model plant, to the soil contaminated with Cu and to determine the effectiveness of the immobilization of this metal through the use of mineral neutralizing materials, such as lime, clay and zeolite.

## 2. Materials and Methods

### 2.1. Experimental Design

The pot experiment was set up in a greenhouse of the University of Warmia and Mazury in Olsztyn (Poland), according to the randomized block method. The experimental factors were (1) increasing soil contamination with copper: 0, 200, 400 and 600 mg Cu kg^−1^ soil; (2) using neutralizing materials (NMs): agricultural lime (98.66% CaCO_3_) (Polcalc, Łódź, Poland) in a dose corresponding to 1 HAC, i.e., 1.06 g CaCO_3_ kg soil^−1^, clay (GEOL-MIN, Kielce, Poland) (clay < 0.002 mm 85.2%, loam 0.05–0.002 mm 13.6%, sand 0.05–2.0 mm 1.2%, d = 2.73 g cm^−3^) and natural zeolite 1.0–2.5 mm (Subio Eko, Sosnowiec, Poland). A Cu stock solution (100 mg Cu cm^−3^) was prepared by dissolving 392.9 g of CuSO_4_·5H_2_O (copper sulfate, pentahydrate) in 1000 cm^3^ (1 dm^3^) of deionized water. The soil in each pot was artificially enriched with the increased concentration of Cu by adding 16, 32, 48 cm^3^ of the Cu pot^−1^ stock solution. Each object along with the control (without enrichment) was carried out in triplicate. Clay and zeolite were applied at a dose of 3% of the weight of the soil in a pot, i.e., 30 g kg of soil^−1^. Before starting the experiment, NPK fertilization was applied: 2.17 g N—CO(NH_2_)_2_, 0.6 g P—KH_2_PO_4_, 1.25 g K—KH_2_PO_4_ (0.75 g K) and K_2_SO_4_ (0.5 g K), 0.18 g Mg—MgSO_4_ and 0.25 mg B—H_3_BO_3_ pot^−1^. Proper brown soil used in the experiment was taken from the Ap level from a field at the Experimental Station in Tomaszkowo located near Olsztyn (53°42’35’’ N, 20°26’01’’ E). The particle size distribution of the soil was 75.1% sand, 24.3% silt and 0.6% clay. The soil texture (according to the USDA) was classified as loamy sand. The basic properties of the soil are presented in Table 1.

The experiment was set up in polyethylene pots filled with 8 kg of soil sieved through a sieve with a mesh diameter of 1 cm. During the vegetative growth, the moisture of the soil in the pots was kept at the level of 60% of full water capacity. Black mustard (*Brassica nigra* L. Koch) of the local population (PPH “PIAST” Jan Kołodziej, Dębno) grown in the pots was harvested after the formation of pods (BBCH 71-79).

### 2.2. Analytical Methods

#### 2.2.1. Soil Analysis

Before setting up the experiment in the starting soil, the particle size distribution was determined using a Mastersizer 3000 (Malvern Instruments Limited, Worcestershire, UK). Then, after sieving through a sieve with a mesh diameter of 2 mm, the soil pH, HAC, TEB, CEC, BS, salinity, N_tot_, TOC, C:N ratio and total copper content (Cu) were determined. Soil analyses were performed using the following methods. Soil pH (pH) was determined potentiometrically in water and in soil 1 M KCl^−1^ solution in the ratio of 1:2.5 (*w*/*v*), using a pH SenTix61 electrode and pH 538 WTW potentiometer (WTW, Wrocław, Poland). EC was determined in a mixture of soil/deionized water in the ratio of 1:2 (*w*/*v*) with a portable multi-range conductivity meter HI-8733 (Hanna Instruments, Leighton Buzzard, United Kingdom). HAC and TEB were determined with the Kappen’s method [38], and the results were used to calculate CEC (1) and BS (2) according to the following formulas:

(1) CEC=HAC+TEB

(2) $$\mathrm{BS}=\frac{\mathrm{TEB}}{\mathrm{CEC}}\times 100$$

The content of N_tot_ was determined by the Kjeldahl method [38], and the total organic carbon (TOC) content was determined on a Shimadzu TOC-L analyzer (Shimadzu Corporation, Kyoto, Japan) coupled with a module SSM-5000A for solid samples. Total Cu content in soil was determined in the samples mineralized according to the US-EPA3051 protocol in a MARS 5 microwave oven (CEM Corporation, Matthews, NC, USA) in a mixture of acids: 65% HNO_3_ and 38% HCl mixed in a 4:1 ratio. The Cu content in soil was determined using an atomic absorption spectrophotometer SpectrAA-240FS (Varian Inc., Mulgrave, Australia), with reference standards by MERCK (Darmstadt, Germany).

#### 2.2.2. Plant Analysis

Before the plants were harvested, the SPAD leaf greenness index was determined with a greenness measuring apparatus (SPAD-502 Chlorophyll Meter, Konica-Minolta, Tokyo, Japan). After harvesting, the yield and the content of Cu, N_tot_, P, K, Ca, Mg and Na were determined. After determining the yield of fresh weight from the pot, the plant samples were fragmented into pieces about 2 cm long and dried at 50 °C in a Binder FED 720 continuous airflow dryer (Binder GMBH, Tuttlingen, Germany) to determine the dry matter content. The samples were ground, and the Cu content was determined after digestion in 65% HNO_3_ acid according to the US-EPA3052 protocol, in a MARS 5 microwave oven. The remaining components were determined after the mineralization of samples in concentrated sulfuric acid, with the addition of hydrogen peroxide as a catalyst. The samples thus prepared were submitted to the following determinations. The content of nitrogen was determined with the Kjeldahl distillation method in a Distillation Unit BUCHI K-355 (BÜCHI Labortechnik AG, Flawil, Switzerland), The vanadium molybdate colorimetric method was used to determine the phosphorus content (P) [38]. Absorbance for P determination was measured at the wavelength of 470 nm in a 1 cm path length quartz cuvette using a flow spectrophotometer Specol 220 (Carl Zeiss Jena, Oberkochen, Germany). The content of potassium (K), calcium (Ca) and sodium (Na) was measured by atomic emission spectrometry (AES), and the content of magnesium (Mg) and copper (Cu) was determined with the flame atomic absorption spectrophotometric method (FAAS) in a VARIAN SpectrAA—FS240 apparatus (Varian Inc., MulgraveAustralia) [38]. Ratios between the nutrients (macroelements) contained in black mustard plants were expressed as equivalent ratios (K, Na, Ca and Mg) and as molar ratios (Ca and P). The equivalent ratios were calculated from the content of the nutrients expressed in g kg^−1^ dm, and their gram equivalent weight (+) (K = 39.098, Na = 22.989, Ca = 20.039 and Mg = 12.153 g val(+)^−1^). Due to the varying valence of phosphorus in chemical compounds, the Ca:P ratios were expressed as molar ones. This is how the Ca:P ratio is presented in the available research papers [39]. This ratio was calculated from the concentrations of Ca and P in plants, expressed in g kg^−1^ DM, and their molar mass (Ca = 40.078, P = 30.972 g mol^−1^). 

#### 2.2.3. Experimental Data Analysis

The results were processed statistically with ANOVA at the level of significance of α ≤ 0.05, using a Statistica^®^ v. 13.3 PL software package from TIBCO Software Inc (Palo Alto, CA, USA) [40]. The correlation between the analyzed factors was established using a simple linear correlation model, with the Microsoft Excel^®^ for Microsoft 365 MSO [41].

## 3. Results and Discussion

### 3.1. Leaf Greenness—SPAD Index

**Figure 1:**
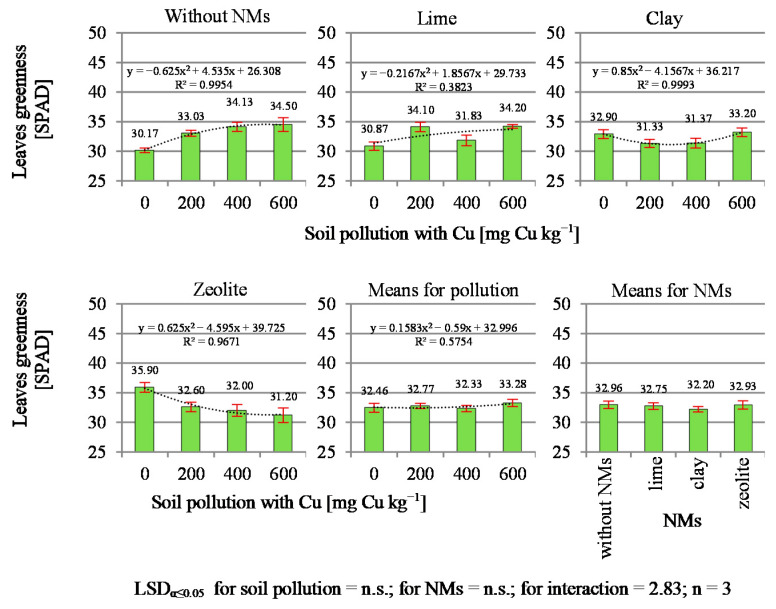
The greenness of the leaves of black mustard (*Brassica nigra* L. Koch).

Soil contamination with copper significantly modified the leaf greenness (SPAD index) of the tested plants (Figure 1). In the series without neutralizing materials, a significant increase in greenness in relation to the control object was shown in objects contaminated with 200 and 400 and 600 mg Cu kg^−1^ of soil. The interaction between the examined factors indicated that the SPAD index reacted differently to the NMs applied in the mentioned objects. First, it was found that a 3% addition of zeolite to uncontaminated soil significantly increased the SPAD index, which was not demonstrated for clay and lime. Comparing the SPAD index of objects with higher copper content in relation to the control object (without NMs), it was found that zeolite completely eliminated the effect caused by the increase in the soil copper concentration, i.e., the chlorophyll content decreased linearly due to the increasing Cu content in the soil. Similar results were obtained in the series with the addition of clay, but only for objects with 200 and 400 mg Cu kg^−1^ of soil. The addition of lime did not bring any significant changes in relation to the control series. 

Leaf greenness index (SPAD) is a reliable indicator for the nutritional status of plants. In precision agriculture, it is used in particular to determine the plant demand for nitrogen fertilization, which is directly correlated with the yield [42,43,44]. The results indicate that SPAD may be useful in the observation of changes in plant pigments (chlorophylls) caused by environmental stress factors, e.g., the influence of heavy metals, salinity, etc. Research on the content of chlorophyll in scentless mayweed plants in the vicinity of a copper smelter conducted by Dopierała [15] showed that, in this case, the chlorophyll content was relatively stable up to the contamination level of 200 mg Cu kg^−1^ of soil, and increasing pollution caused a slight decrease in greenness. The present research showed that 200 mg Cu kg^−1^ as a stressor was already high enough to induce an increase in the content of chlorophyll in leaves. A similar effect of increasing the content of chlorophyll under the influence of copper was shown by Feil et al. [45]. The authors, using low concentrations of copper (5 μM Cu dm^−3^) in the medium in which cucumbers were grown, showed a decrease in the SPAD index by 0.5 units compared to the control object (0.2 μM Cu dm^−3^), while in higher concentrations of Cu (25 and 50 μM Cu dm^−3^), the SPAD index increased by 1.7 and 9.3 units. Disruptions in the biosynthesis of chlorophyll are one of the most negative consequences resulting from the toxicity of copper for plants [12]. High soil contamination with copper results in reduced plant growth and leaf chlorosis [33], and as a result of the formation of reactive oxygen species (ROS), it contributes to oxidative stress and thus disrupts metabolic pathways and causes damage to macromolecules [46,47,48]. The presented data indicate that increasing the content of minerals such as zeolite and clay in copper-contaminated sites may significantly modify the content of chlorophyll in leaves of cultivated plants.

### 3.2. Plant Yield and Dry Matter Content

**Figure 2:**
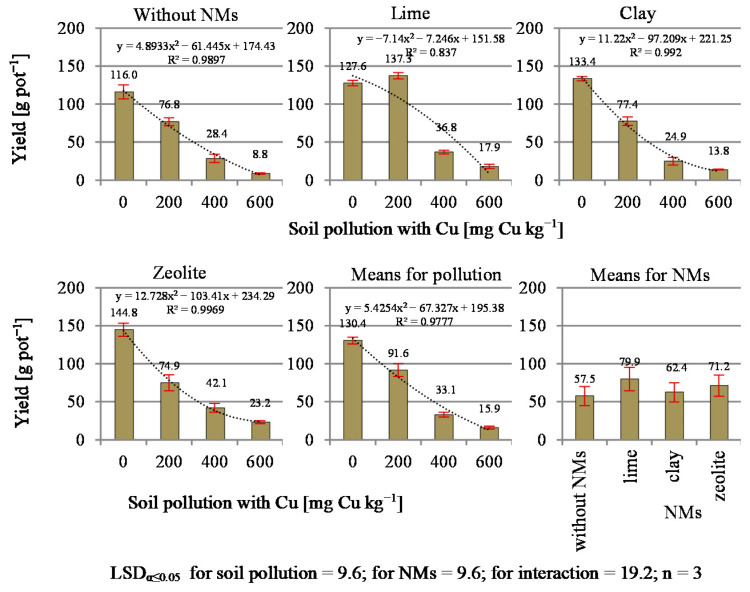
The yield of aerial mass of black mustard (*Brassica nigra* L. Koch).

The high toxicity of Cu manifested by the above-mentioned disturbances in the synthesis of chlorophyll has a direct impact on the biomass yield of plants. In the present research, the black mustard yield (Figure 2) depended on both soil contamination and the applied NMs. As the level of contamination increased, the yield decreased significantly. In the series without NMs, at the level of 200 mg Cu kg^−1^, this decrease was on average 33.8% in relation to the fresh weight of plants in the control sample, while contamination at the higher levels of 400 and 600 mg Cu kg^−1^ resulted in a reduction of the yield by 75.5% and 92.4%, respectively. The NMs significantly influenced the differentiation of the results in relation to the control series without NMs. The use of lime significantly reduced the toxicity of copper, even effecting an increase in the plant yield in relation to the yield obtained in the control object. It was most visible for the level of 200 mg Cu kg^−1^, where, in relation to the series without NMs, the increase in the yield amounted to 7.6% in relation to the control. With regard to the higher concentrations of copper in the soil, no mitigating effect of lime was demonstrated. The remaining NMs did not reduce the harmful effects of copper. In the case of zeolite, the mitigating effect on copper was observed in objects contaminated with Cu at the level of 400 mg kg^−1^ and 600 mg kg^−1^. However, this effect was not statistically proven, but the yields obtained in this series, compared to those harvested in the series without NMs, were higher by 13.7 and 14.4 g pot^−1^, respectively. The results indicate that the Cu content in the soil at the level of 200 mg kg^−1^ results in a decrease in plant biomass yield. The toxic effect of Cu on plants, reflected in reduced yield, was demonstrated in the research by Xu et al. [49]. In the cultivation of rice, at the levels of copper contamination used by the authors, yield dropped by 10.1% for 100 mg Cu kg^−1^, 15.4% for 200 mg Cu kg^−1^, 37.0% for 400 mg Cu kg^−1^, 8.9% for 600 mg Cu kg^−1^, 89.3% for 800 mg Cu kg^−1^ and 96.2% for 1000 mg Cu kg^−1^ compared to unpolluted objects. Among the NMs used in the present research, lime significantly reduced the toxic effect of copper, even causing an increase in the yield in the object with 200 mg Cu kg^−1^. The addition of NMs improved the use of the soil nutrients and fertilizers by plants. According to Plyatsuk [2] and Derakhshan Nejad et al. [50], lime has a significant effect on the soil microflora. In addition, the ability of roots to absorb a number of heavy metals, in particular lead, decreases as a result of an increase of the Ca content in the soil and more intensive encrustation of the root cell walls with these components. Liming also contributes to the formation of complexes of soil organic substances with metals, which reduces the mobility of Cu and Hg, Cd, Zn, Ni and Cr. 

**Figure 3:**
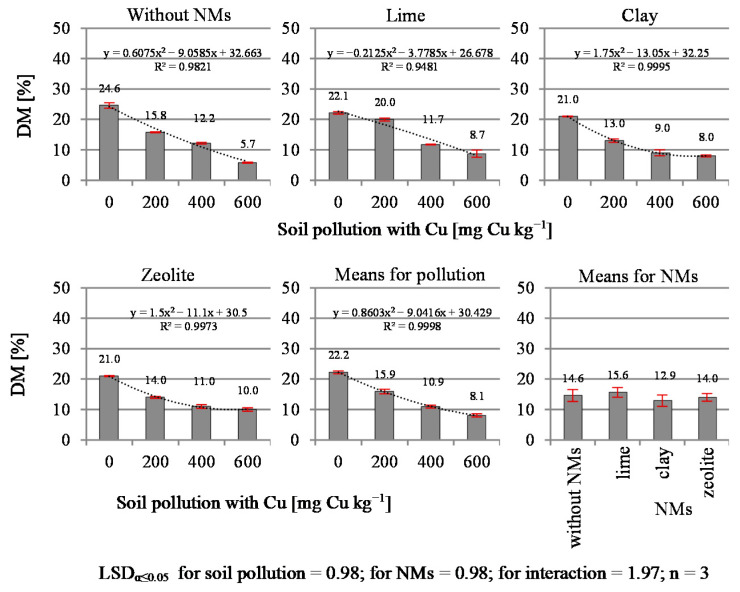
Content of dry mass in black mustard (*Brassica nigra* L. Koch).

The dry matter (DM) content in the test plant (Figure 3) was highly dependent on the increasing level of soil contamination with copper and the applied NMs. Along with the increase in soil contamination, a decrease in the dry matter content was observed. The greatest decrease in DM content (36.0% on average) occurred between 0 and 200 mg Cu kg^−1^. The demonstrated interaction suggests that the NMs modified the DM content in different ways at certain levels of soil contamination with copper. Similarly to its effect on yield, the addition of lime applied in the object contaminated with 200 mg Cu kg^−1^ significantly increased the DM content of plants compared to the control object, i.e., without NMs, and those where clay or zeolite were used. A similar effect after the application of lime was found in a study on neutralization of the toxic effect of copper on maize [7]. 

In the case of this plant, the addition of lime used in objects contaminated with copper at the level of 200 mg Cu kg^−1^ resulted in an increase in the dry matter content in relation to the control sample. These results correspond with the research by Szatanik-Kloc [51], who showed a stressful effect of copper on plants in trials with increasing copper contamination, resulting in a decrease in the dry matter content of plants compared to the control sample. In addition, varied responses of different plant species to copper contamination have been demonstrated. For example, wheat and clover were more susceptible to stress than rye and lupines.

A previous study conducted by Żołnowski et al. [7] showed that a dose of 200 mg Cu kg^−1^ of soil had a stimulating effect, increasing the maize root yield in relation to the control object. The authors did not find such an action in relation to the aerial mass of maize. The present research has shown that in the case of black mustard sown in sites where copper contamination reaches up to 200 mg Cu kg^−1^ soil, lime is a good neutralizer, limiting the element’s toxic effect. For higher copper concentrations, zeolite is a better alternative.

### 3.3. Chemical Composition of Plants

The Cu content in plants was varied and determined by the Cu content in the soil (Table 2). In all series, it was shown that the Cu content was significantly affected by the level of 200 mg Cu kg^−1^ of soil. The tendency for a further increase in the copper content in the tissues of black mustard was also demonstrated in relation to the level of 400 mg Cu kg^−1^ of soil in the series without NMs and with the addition of lime and of clay. In the series with the addition of zeolite, a tendency to decrease the copper content was demonstrated under the influence of 400 mg Cu kg^−1^ of soil. The dose of 600 mg Cu kg^−1^ of soil in each of the tested objects caused a decrease in the copper content in relation to objects contaminated with 400 mg Cu kg^−1^ of soil. The observed decrease in Cu content could have been conditioned by the plant defense mechanism. There are a number of mechanisms that can reduce the stressful effects of metals on plant growth. Among them, there is the mechanism of metal ion sequestration through their accumulation and inactivation in forms related to organic and inorganic components contained in cells, e.g., oxalates or phenolic compounds [28,52]. With an excess of harmful ions in the cytoplasm, the plant can integrate them into the structure of cell walls through secondary transport [48]. It can be assumed that the present research revealed this model of a detoxification mechanism. Pedersen et al. [53], investigating the content of copper in black bindweed biomass, mentioned a similar phenomenon. The authors found that the accumulation of copper in shoots increased up to total soil copper concentrations of 300–400 mg Cu kg^−1^ and then stabilized at higher soil copper concentrations (500 and 600 mg Cu kg^−1^). Similarly, Amin et al. [54] observed an increase in the Cu content in plants (*Abelmoschus esculentus*, *Avena sativa*, *Guizotia abyssinica*, and *Glycine max*) to a dose of 300 mg Cu kg^−1^. 

There are a number of studies confirming that plant roots are the first organ in which the excess of harmful metals is sequestered [33,50,55,56]. This is especially the case for non-hyperaccumulator plants. Hyperaccumulators rapidly and efficiently translocate toxic metals from roots to shoots via the xylem [57]. Żołnowski et al. [7], examining the content of copper in maize, found that a much greater amount of this element was sequestered in the roots than in the aerial parts of plants. 

The copper content of plants can vary within very wide limits. Factors influencing the amount of Cu accumulation include plant species, cultivar and development stage. Plants collected from areas with a high content of heavy metals belong to two groups, i.e., tolerant plants and hyperaccumulators. The latter, as reported by Van Der Ent et al. [58], can accumulate Cu in amounts from >300 to >1000 mg kg^−1^ DM. Generally, the Cu content in standard reference plants has an elemental concentration of 10 mg kg^−1^ DM. For most plants (also crops), the content of 20 mg kg^−1^ DM is already toxic [17]. 

Soil liming is a factor that significantly limits copper uptake. Among the NMs used in the present research, only lime significantly reduced the accumulation of copper in black mustard plants. Compared to the control series, a decrease in the content of this metal by 114 mg Cu kg^−1^ DM was demonstrated. The effect of limiting the lability of this metal could be directly related to the strong blockage of copper in the soil in the form of water-insoluble salts under conditions of lower soil acidity due to the use of lime. The present research is consistent with the results of Żołnowski et al. [7], where the addition of lime significantly reduced the Cu content in the dry matter of maize, especially at the contamination levels of 400 and 600 mg Cu kg^−1^. It was found that the copper content decreased by 59.4% and 43.8% in the dry matter of roots and by 67.6% and 56.3% in the dry matter of the aerial part of maize compared to the series without liming. Ca has been shown to decrease heavy-metal-induced toxicity [59,60]. In the case of lime, a particularly significant mitigating effect of copper is observed in acidic soils. Antoniadis and Damalidis [61] showed that 100 days after the application of 6.6 Mg of lime ha^−1^ to soil with pH = 3.8, the Cu concentration in plants was 21.0% lower compared to the objects without liming. At the same time, the authors emphasize the significant influence of zeolite, which, when applied at a dose of 3.3–10.0% of the soil mass on the 50th day of the research, was more effective than lime in reducing copper uptake. As a result, the authors showed that zeolite used with lime reduced the Cu concentration by 43.0%. In the present study, the soil was characterized by much higher pH (pH_KCl_ = 6.44), at which the effect demonstrated by Antoniadis and Damalidis [61] was not observed after the application of zeolite. 

The content of total nitrogen (N_tot_) (Table 3) in black mustard yield was significantly differentiated and depended on the level of copper contamination and the NMs used. The increasing pollution stimulated the accumulation of nitrogen compounds (up to 400 mg Cu kg^−1^). The applied lime decreased the amount of nitrogen accumulation in the plant compared to the control series, while the NMs in the form of clay and zeolite increased the N_tot_ content in the test plant. The increase in the content of N_tot_ in plants basically occurred only up to the level of 400 mg Cu kg^−1^. The pollution with 600 mg Cu kg^−1^ decreased the N_tot_ content in plant tissue.

The content of minerals in the yield of plants depends on the efficiency of nutrient uptake. The uptake depends on the mechanism of ion transportation from the soil solution to the root and further through the cells of the tissues conducting to the stem, leaves, flowers and fruits. It takes place through the root cell membranes, with the participation of the so-called transport proteins [62]. This transport is effective up to a specific (borderline) content of the component in the soil, above which problems resulting from soil salinity most often occur. In contaminated soils, the component limiting the uptake of nutrients, and thus the content of elements in plants, is considered to be a contaminant. With regard to the content of copper in soil and nitrogen in plant biomass, there are a number of studies confirming the synergism between these elements. Such results were obtained by Kuziemska et al. [63]. In that research, the nitrogen content of cocksfoot grass biomass increased in the first year of cultivation as a result of the increasing copper content in the soil (100–300 mg Cu kg^−1^). In addition, Snowball et al. [64], with reference to subterranean clover, and Alhasany et al. [65], in relation to broad bean, reported a synergistic effect of these two elements. On the other hand, the research of Meller and Jarnuszewski [66] shows no relationship between copper fertilization and the N content in the biomass of maize, spring barley, spring wheat, white mustard, spring oilseed rape and common oat. The data published by Rietra et al. [62] also indicate the lack of antagonistic and synergistic interactions between copper and nitrogen. 

In this study, a synergistic effect of Cu on the uptake and N_tot_ content in black mustard biomass up to the dose of 400 mg Cu kg^−1^ of soil was observed. After exceeding this level of soil contamination with Cu, an antagonistic effect of this metal in relation to N was observed.

Along with the increase in the soil contamination with copper, a decrease in the P content in plants was shown (Table 3). The lowest P content was found for 600 mg Cu kg^−1^ soil. The applied NMs did not significantly change the P content in black mustard plants, although a noticeable decrease in the P content was observed in each series under the influence of increasing contamination. The highest average content of P was found in the series with the addition of lime (24.1% higher than in the series without neutralizing agents). The antagonistic effect of Cu on the P content was confirmed by Feil et al. [45]. Cucumber plants treated with the highest concentration of Cu showed, significantly, the lowest concentration of P in both shoots and roots, decreased by approximately 32 and 36.0%, respectively, compared to the control. In addition, Szatanik-Kloc [51] found in her research the antagonism of Cu against P in tissues of clover, wheat and rye. According to the author, reduction of the P content in plant tissues is most often associated with a change in the permeability of cell membranes, which are responsible for the transport of ions such as PO_4_^3−^ or K^+^ [17].

Soil contamination with copper significantly influenced the potassium content in plants (Table 3). The highest content of this element in plants in most of the experimental series was found at the Cu pollution equal to 400 mg kg^−1^ of soil. In the series without NMs, it was 42.25 g K kg^−1^ DM, and this value was more than twice as high as in the control sample. The highest soil contamination with copper, 600 mg Cu kg^−1^, resulted in a decrease in the K content by 14.1%, which was probably related to toxic effect. All the NMs caused a decrease in the mean potassium content compared to the control object without NMs. The potassium content in plants was lowered the most by clay, then by zeolite and lime.

Changes in the content of Ca, Mg and Na, as in the case of K, differed depending on the increasing level of pollution and the NMs used (Table 4). The content of these elements in the control series increased to the pollution level of 400 mg Cu kg^−1^. At the level of 600 mg kg^−1^, a decrease in the content of these elements was observed. The applied NMs in the form of lime and zeolite contributed to an increase in the Ca content, which was not demonstrated for clay. Concerning Mg, its decrease was noted after the use of clay and lime. The average Na content was stimulated by the use of zeolite, whereas lime and clay significantly reduced the content of this element in plants. Summing up, soil contamination with Cu up to the level of 400 mg kg^−1^ contributed to an increase in the content of potassium, calcium, magnesium and sodium. At higher pollution, i.e., 600 mg Cu kg^−1^, a decrease in the content of these macronutrients was observed. A decrease in the content of calcium and magnesium in plants correlating with an increase in copper contamination was also confirmed in the studies by Szatanik-Kloc [51] and Alaouis-Sosse et al. [67].

### 3.4. Ion Balance

The quality of plants is expressed by both the total content of macronutrients and their mutual relations. Ionic ratios in plants are highly correlated with soil nutrient abundance. The inflow of various mineral and organic pollutants to the soil, through their synergism and antagonism, may significantly disturb the ionic balance of soils and thus directly affect the conditions of growth and development as well as the chemical composition of plants. Under production conditions, the analysis of this composition may indicate which components should be given particular attention when fertilizing plants. The ionic ratios in plants provided in scientific studies usually concern fodder plants [68,69]. Older works by other authors cited in these publications indicate that the optimal values of the ratios between individual ions in plants, expressed in milligram equivalents, are as follows: K:(Ca+Mg) 1.6–2.2:1; K:Mg 6:1; K:Ca 2:1; K:Na 5–10:1; Ca:Mg 2–3:1; K:Ca 2:1 and the molar ratio of Ca:P 2:1. 

**Figure 4:**
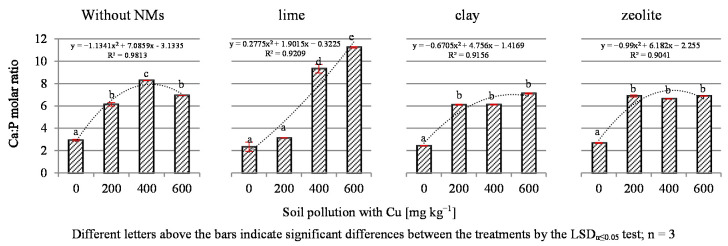
The molar Ca:P ratio in dry mass of tissue of black mustard (*Brassica nigra* L. Koch).

The present study showed that increasing soil contamination with copper contributed to a significant extension of the Ca:P molar ratio compared to the values adopted for forage plants (Figure 4). 

This ratio should be about 2:1; meanwhile, the increasing Cu content in the soil increased this ratio to 6.14:1, 8.28:1 and 6.94:1 for 200, 400 and 600 mg Cu kg^−1^ soil, respectively. The applied NMs did not significantly change the trend of the Ca:P ratio, with the exception of lime, which in response to 400 and 600 mg Cu kg^−1^ soil additionally increased the already high values of these ratios to 10.33:1 and 11.24:1. The demonstrated broad Ca:P ratios can be associated with excessively low calcium content in plants. However, comparing the content of these elements in the black mustard samples to the average values in cultivated plants, which is in the range of 2–5 mg P kg^−1^ DM [38], and the tendency to change the P content under the influence of copper, it should be noted that the expansion of the Ca:P ratio is due to the decrease in the P content caused by the harmful effects of Cu.

**Figure 5:**
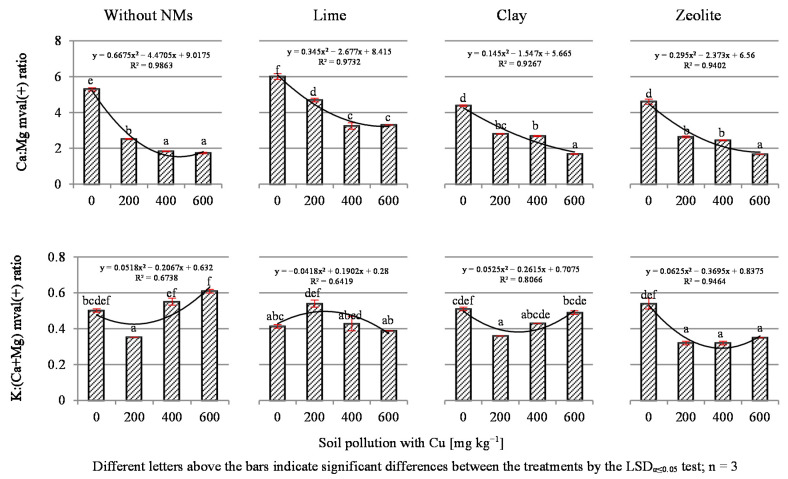
The Ca:Mg and K:(Ca+Mg) ratio in dry mass of tissue of black mustard (*Brassica nigra* L. Koch).

Soil contamination with Cu significantly influenced the Ca:Mg ratio in the test plant (Figure 5). In the control series, the narrowing of this ratio was observed from 5.29:1 for controls to 2.52:1 and 1.84:1 for 200 and 400 mg Cu kg^−1^, respectively. Lime significantly widened the Ca:Mg ratio compared to the series without NMs, while the remaining mineral NMs did not significantly affect the formation of this indicator.

The K:(Ca+Mg) ratio in the analyzed mustard plants was narrowed under the influence of lime, but the other NMs did not modify this parameter significantly (Figure 5). It was found that the K:(Ca+Mg) ratio was widened in the objects with 200–600 mg Cu kg^−1^, but the value of 2.2 was never exceeded. The K:(Ca+Mg) ratio higher than 2.2 in animal feed may cause symptoms of tetany [34,35,69].

**Figure 6:**
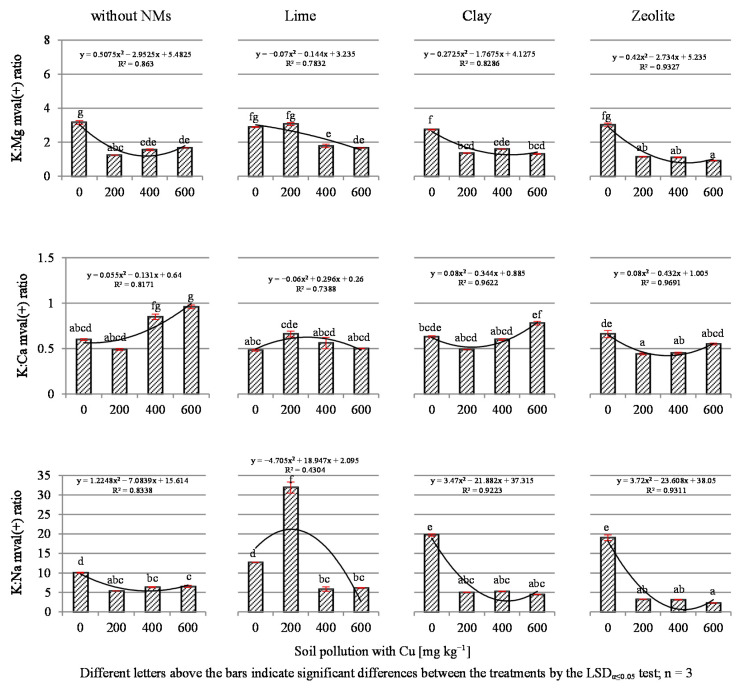
The K:Mg, K:Ca and K:Na ratios in dry mass of tissue of black mustard (*Brassica nigra* L. Koch).

The remaining equivalence ratios between individual ions, presented in Figure 6, developed in different ways and depended on the assessed elements, Cu contamination and the NMs used. With regard to the K:Mg, K:Ca and K:Na ratios, it was shown that Cu contamination generally led to the narrowing of these values. The results seem to confirm the significant narrowing of the K:Ca and K:Mg ratios under the influence of copper as observed by Jarnuszewski and Meller [70], who showed such changes in the straw of spring barley under the application of Cu as a micronutrient fertilizer. It should be stated, however, that the opposite tendency was observed in white mustard, spring oilseed rape and common oat, which indicates species-specific differences between agricultural plants with regard to the response to soil contamination with copper.

## 4. Conclusions

In the present research, copper as a soil polluting factor influenced the yields and chemical composition of the plants obtained. The influence of Cu on the greenness of leaves became evident during the growing season. Cu stimulated plants to increase the production of chlorophyll to the level of 400 mg Cu kg^−1^ soil. The addition of zeolite to soil with 400 mg Cu kg^−1^ of soil and or clay to soil exposed to the entire range of simulated contamination counteracted these changes.

Due to the increasing content of copper in the soil, the yield of plants and the dry matter content decreased sharply. The NMs mitigated this harmful influence, with clay, zeolite and lime in the increasing order of the mitigation effect. In the lime in objects contaminated with 200 mg Cu kg^−1^ of soil, an increase in black mustard yield was observed in relation to the control.

Under the influence of soil contamination with Cu, a linear increase in Cu concentration in plants was observed, with the maximum accumulation achieved at the contamination level of 400 mg Cu kg^−1^ of soil. Above this level, a decrease in the Cu content in the aerial parts of plants was demonstrated. This effect could have been caused by the activation of copper sequestration mechanisms in the root tissue. The content of K, Mg, Ca and Na in plants increased to the level of 400 mg Cu kg^−1^, while decreasing above this level, while the content of P decreased in proportion to the increasing pollution. Lime reduced the Cu accumulation in black mustard at all levels of contamination. Clay reduced the copper content in the objects with lower pollution (200 and 400 mg Cu kg^−1^) and in the one not contaminated with Cu relative to the control series. 

Copper, through interaction with other components, favored the narrowing of the Ca:Mg, K:Mg and especially K:Na ratios and the expansion of the Ca:P and K:Ca ratios in plants. An unexpected exception was the object contaminated with 200 mg Cu kg^−1^ fertilized with lime, where the value of K:Na increased to 31.90:1. Lime contributed to the extension of the Ca:P ratio, especially in objects contaminated with 400 and 600 mg Cu kg^−1^. Lime also acted positively by expanding the Ca:Mg ratio compared to the series without NMs.

The most beneficial mineral raw material mitigating the effects of copper contamination was lime, followed by zeolite (especially at high copper concentrations). 

## Figures and Tables

**Table 1:** Selected physicochemical properties of soil used in experiment.

Soil Type	pH KCl	pH H2O	HAC [cmol kg−1]	TEB [cmol kg−1]	CEC [cmol kg−1]	BS [%]	Salinity [μS cm−1]	Ntot [g kg−1]	TOC [g kg−1]	C:N ratio	Cu [mg kg−1]
Cambisols—Brown Soil 1	6.44	7.07	2.46	6.57	9.03	72.75	0.89	0.58	4.79	8.26	18.21

HAC: hydrolytic acidity; TEB: total exchangeable bases; CEC: cation exchange capacity; BS: base saturation; N_tot_: total nitrogen; TOC: total organic carbon; ^1^ IUSS Working Group WRB [37].

**Table 2:** Copper content in black mustard (*Brassica nigra* L. Koch).

Soil Pollution with Cu (mg kg^−1^)	Without NMs		Neutralizing Materials						Mean	
			Lime		Clay		Zeolite			
Copper content (mg Cu kg^−1^ DM)										
0	76.97 ± 15.95	^b^	16.48 ± 4.17	^a^	22.02 ± 4.70	^a^	74.54 ± 2.12	^b^	47.50 ± 9.25	^A^
200	371.18 ± 12.72	^ef^	222.05 ± 6.66	^c^	356.40 ± 0.00	^de^	424.22 ± 5.28	^f^	343.46 ± 21.84	^C^
400	398.18 ± 4.66	^ef^	244.59 ± 13.97	^c^	379.69 ± 1.74	^ef^	383.38 ± 3.48	^ef^	351.46 ± 18.32	^C^
600	310.29 ± 7.97	^d^	217.41 ± 12.20	^c^	346.67 ± 8.04	^de^	368.65 ± 5.78	^ef^	310.76 ± 17.25	^B^
Mean:	289.15 ± 36.97	^BC^	175.13 ± 27.08	^A^	276.19 ± 45.57	^B^	312.70 ± 40.19	^C^	263.29	
r	0.63	^ns^	0.74	*	0.76	*	0.68	^ns^	-	
LSD α ≤ 0.05 for increasing Cu soil pollution = 26.08; for amendments = 26.08; for interaction = 52.15										

Means followed by different letters are significantly different by the LSD_α__≤0_._05_ test; *—correlation coefficient *r* significant for α ≤ 0.05; ^ns^—non significant; n = 3.

**Table 3:** Total nitrogen, phosphorus and potassium content in black mustard (*Brassica nigra* L. Koch).

Soil Pollution with Cu (mg kg^−1^)	Without NMs		Neutralizing Materials						Mean	
			Lime		Clay		Zeolite			
Ntot (g kg^−1^ DM)										
0	9.88 ± 0.46	^a^	13.30 ± 0.07	^b^	21.70 ± 0.07	^d^	21.84 ± 0.13	^d^	16.68 ± 1.51	^A^
200	28.56 ± 0.00	^e^	19.46 ± 0.07	^c^	36.40 ± 0.13	^g^	39.48 ± 0.26	^ij^	30.98 ± 2.24	^B^
400	36.26 ± 0.07	^g^	32.62 ± 0.07	^f^	42.28 ± 0.66	^l^	41.30 ± 0.07	^kl^	38.12 ± 1.14	^D^
600	37.38 ± 0.07	^gh^	32.76 ± 0.00	^f^	40.32 ± 0.13	^jk^	38.78 ± 0.20	^hi^	37.31 ± 0.82	^C^
Mean:	28.02 ± 3.18	^B^	24.54 ± 2.44	^A^	35.18 ± 2.33	^C^	35.35 ± 2.27	^C^	30.77	
r	0.92	**	0.95	**	0.85	**	0.75	*	-	
LSDα≤0.05 for increasing Cu soil pollution = 0.73; for NMs = 0.73; for interaction = 1.46										
P (g kg^−1^ DM)										
0	3.89 ± 0.04		5.93 ± 1.07		4.10 ± 0.04		3.95 ± 0.01		4.47 ± 0.36	^B^
200	3.01 ± 0.08		4.02 ± 0.02		3.22 ± 0.00		3.09 ± 0.02		3.34 ± 0.12	^B^
400	2.38 ± 0.01		2.62 ± 0.10		3.05 ± 0.00		2.80 ± 0.00		2.71 ± 0.07	^AB^
600	2.15 ± 0.03		2.01 ± 0.01		2.06 ± 0.00		2.32 ± 0.02		2.14 ± 0.04	^A^
Mean:	2.86 ± 0.20		3.64 ± 0.51		3.11 ± 0.21		3.04 ± 0.17		3.16 ± 0.04	
r	−0.96	**	−0.78	*	−0.97	**	−0.97	**		
LSDα≤0.05 for increasing Cu soil pollution = 0.86; for NMs = ns; for interaction = ns										
K (g kg^−1^ DM)										
0	17.14 ± 0.12	^ab^	14.41 ± 0.23	^a^	15.88 ± 0.24	^ab^	17.70 ± 0.86	^ab^	16.28 ± 0.43	^A^
200	22.91 ± 0.28	^bc^	20.98 ± 1.19	^abc^	24.09 ± 0.00	^bc^	23.51 ± 0.56	^bc^	22.87 ± 0.48	^B^
400	42.25 ± 1.27	^f^	34.56 ± 3.79	^de^	28.11 ± 0.15	^cd^	21.20 ± 0.53	^abc^	31.53 ± 2.46	^C^
600	36.29 ± 1.18	^ef^	28.75 ± 0.15	^cde^	28.76 ± 0.46	^cde^	22.33 ± 0.00	^abc^	29.03 ± 1.46	^C^
Mean:	29.65 ± 2.94	^B^	24.68 ± 2.42	^A^	24.21 ± 1.49	^A^	21.19 ± 0.69	^A^	24.93	
r	0.84	**	0.73	*	0.92	**	0.52	^ns^	-	
LSDα≤0.05 for increasing Cu soil pollution = 3.60; for NMs = 3.60; for interaction = 7.21										

Means followed by different letters are significantly different by the LSD_α__≤0_._05_ test; *—correlation coefficient *r* significant for α ≤ 0.05; **—correlation coefficient *r* significant for α ≤ 0.01; ^ns^—non significant; n = 3.

**Table 4:** Calcium, magnesium and sodium content in black mustard (*Brassica nigra* L. Koch).

Soil Pollution with Cu (mg kg^−1^)	Without NMs		Neutralizing Materials						Mean	
			Lime		Clay		Zeolite			
Ca (g kg^−1^ DM)										
0	14.76 ± 0.18	^bc^	15.34 ± 0.06	^c^	12.98 ± 0.04	^a^	13.79 ± 0.26	^ab^	14.22 ± 0.27	^A^
200	23.82 ± 0.01	^g^	16.36 ± 0.12	^d^	25.43 ± 0.21	^h^	27.51 ± 0.17	^i^	23.28 ± 1.22	^C^
400	25.51 ± 0.16	^h^	31.39 ± 0.04	^k^	24.14 ± 0.17	^g^	24.05 ± 0.09	^g^	26.27 ± 0.87	^D^
600	19.36 ± 0.23	^e^	29.19 ± 0.07	^j^	18.97 ± 0.14	^e^	20.68 ± 0.22	^f^	22.05 ± 1.21	^B^
Mean:	20.86 ± 1.21	^A^	23.07 ± 2.10	^C^	20.38 ± 1.42	^A^	21.51 ± 1.47	^B^	21.46	
r	0.41	^ns^	0.87	**	0.38	^ns^	0.38	^ns^	-	
LSDα≤0.05 for increasing Cu soil pollution = 0.49; for amendments = 0.49; for interaction = 0.99										
Mg (g kg^−1^ DM)										
0	1.70 ± 0.05	^a^	1.55 ± 0.03	^a^	1.80 ± 0.01	^a^	1.82 ± 0.02	^a^	1.72 ± 0.03	^A^
200	5.74 ± 0.05	^bc^	2.12 ± 0.06	^a^	5.50 ± 0.03	^b^	6.33 ± 0.08	^cd^	4.92 ± 0.48	^B^
400	8.39 ± 0.02	^f^	5.95 ± 0.32	^bc^	5.46 ± 0.02	^b^	5.96 ± 0.03	^bc^	6.44 ± 0.34	^C^
600	6.75 ± 0.22	^d^	5.34 ± 0.02	^b^	6.83 ± 0.00	^d^	7.52 ± 0.07	^e^	6.61 ± 0.24	^C^
Mean:	5.64 ± 0.72	^C^	3.74 ± 0.56	^A^	4.90 ± 0.54	^B^	5.41 ± 0.62	^C^	4.92	
r	0.80	*	0.87	**	0.90	**	0.87	**	-	
LSDα≤0.05 for increasing Cu soil pollution = 0.33; for amendments = 0.33; for interaction = 0.67										
Na (g kg^−1^ DM)										
0	1.00 ± 0.02	^c^	0.66 ± 0.01	^b^	0.47 ± 0.00	^a^	0.55 ± 0.01	^ab^	0.67 ± 0.06	^A^
200	2.51 ± 0.03	^d^	0.39 ± 0.00	^a^	2.81 ± 0.01	^e^	4.23 ± 0.06	^j^	2.49 ± 0.40	^B^
400	3.90 ± 0.05	^h^	3.45 ± 0.03	^g^	3.12 ± 0.00	^f^	3.95 ± 0.03	^i^	3.61 ± 0.10	^C^
600	3.27 ± 0.04	^i^	2.74 ± 0.02	^e^	3.75 ± 0.20	^h^	5.66 ± 0.03	^k^	3.86 ± 0.32	^D^
Mean:	2.67 ± 0.31	^C^	1.81 ± 0.38	^A^	2.54 ± 0.36	^B^	3.60 ± 0.54	^D^	2.66	
r	0.84	**	0.79	*	0.91	**	0.90	**	-	
LSDα≤0.05 for increasing Cu soil pollution = 0.09; for amendments = 0.49; for interaction = 0.18										

Means followed by different letters are significantly different by the LSD_α__≤0_._05_ test; *—correlation coefficient *r* significant for α ≤ 0.05; **—correlation coefficient *r* significant for α ≤ 0.01; ^ns^—non significant; n = 3.

## Data Availability

Data are available by contacting the authors.
